# Efficacy and safety of Elian Granules in treating chronic atrophic gastritis: study protocol for a randomized, double-blind, placebo-controlled, multicenter clinical trial

**DOI:** 10.1186/s13063-022-06395-6

**Published:** 2022-05-24

**Authors:** Zhijian Gu, Qingling Jia, Jun Cong, Rong Cen, Yongqi Chen, Chenheng Wu, Biao Gong, Xudong Tang, Jianghong Ling

**Affiliations:** 1 Department of Gastroenterology, Shuguang Hospital affiliated with Shanghai University of Traditional Chinese Medicine, 185 Pu’an Road, Huangpu District, Shanghai, 200021 China; 2 Endoscopy Center, Shuguang Hospital affiliated with Shanghai University of Traditional Chinese Medicine, Shanghai, China; 3 Department of Pathology, Shuguang Hospital affiliated with Shanghai University of Traditional Chinese Medicine, Shanghai, China; 4 Institute of Spleen and Stomach Diseases, Xiyuan Hospital of China Academy of Chinese Medical Sciences, Beijing, China

**Keywords:** Chronic gastritis, Chronic atrophic gastritis, Elian Granules, Traditional Chinese medicine, Randomized controlled trial

## Abstract

**Background:**

Multifocal atrophic gastritis and intestinal metaplasia are considered to be important links in the gastric precancerous cascade. However, there are no specific drugs for these conditions. Although many studies have shown that traditional Chinese medicine is effective with no serious side effects, these studies have not been scientifically rigorous trials. Our aim is to design a high-quality trial for a Chinese patent medicine, Elian Granules, to investigate its efficacy and safety in treating patients with chronic atrophic gastritis with or without intestinal metaplasia.

**Methods:**

This is a phase II, randomized, double-blind, placebo-controlled, multicenter clinical trial. A total of 240 participants will be assigned to a treatment or placebo control group in a 1:1 ratio. The experimental drug or placebo will be taken with boiling water, two small bags (24.2 g) each time, twice a day, half an hour after a meal, for 24 weeks. The primary outcome is the observation of histological changes in the gastric mucosa of patients with atrophic gastritis with or without intestinal metaplasia after 6 months based on the OLGA/OLGIM staging systems. The secondary outcomes include the assessment of dyspepsia and quality of life based on the dyspepsia symptom score and the quality-of-life scale.

**Discussion:**

This study is designed to evaluate the efficacy and safety of Elian Granules in a randomized, double-blind, placebo-controlled, multicenter manner. This trial may not only provide evidence for a phase III clinical trial, but also an alternative option for the treatment of chronic atrophic gastritis (CAG).

**Trial registration:**

Registry Platform For Evidence-Based Traditional Chinese Medicine ChiMCTR2000003929. Registered on 13 September 2020

## Administrative information

Note: the numbers in curly brackets in this protocol refer to SPIRIT checklist item numbers. The order of the items has been modified to group similar items (see http://www.equator-network.org/reporting-guidelines/spirit-2013-statement-defining-standard-protocol-items-for-clinical-trials/).

**Table:** 

Title {1}	Efficacy and safety of Elian Granules in treating chronic atrophic gastritis: study protocol for a randomized, double-blind, placebo-controlled, multicenter clinical trial
Trial registration {2a and 2b}	ChiMCTR2000003929 [http://www.ccebtcm.org.cn] [registered on 13 September 2020]
Protocol version {3}	13-9-2020, Version 1.3
Funding {4}	National Administration of Traditional Chinese Medicine: 2019 Project of building evidence based practice capacity for TCM (No. ZZ13-042-2, No. 2019XZZX-XH013).
Author details {5a}	Zhijian Gu: Department of Gastroenterology, Shuguang Hospital affiliated with Shanghai University of Traditional Chinese Medicine Qingling Jia: Department of Gastroenterology, Shuguang Hospital affiliated with Shanghai University of Traditional Chinese Medicine Jun Cong: Department of Gastroenterology, Shuguang Hospital affiliated with Shanghai University of Traditional Chinese Medicine Rong Cen: Endoscopy center, Shuguang Hospital affiliated with Shanghai University of Traditional Chinese Medicine Yongqi Chen: Department of Pathology, Shuguang Hospital affiliated with Shanghai University of Traditional Chinese Medicine Chenheng Wu: Department of Gastroenterology, Shuguang Hospital affiliated with Shanghai University of Traditional Chinese Medicine Biao Gong: Department of Gastroenterology, Shuguang Hospital affiliated with Shanghai University of Traditional Chinese Medicine Xudong Tang: Institute of Spleen and Stomach Diseases, Xiyuan Hospital of China Academy of Chinese Medical Sciences Jianghong Ling: Department of Gastroenterology, Shuguang Hospital affiliated with Shanghai University of Traditional Chinese Medicine
Name and contact information for the trial sponsor {5b}	Jianghong Ling (Principal Investigator) ljh18817424778@163.com
Role of sponsor {5c}	This is an investigator-initiated clinical trial. Therefore, the funders play no role in the design of the study and collection, analysis, and interpretation of the data and the writing of the manuscript.

## Introduction

### Background and rationale {6a}

Despite the declining incidence of gastric cancer, it remains one of the most common and lethal malignant tumors worldwide, especially in Asian countries [1]. This is closely related to the high prevalence of *Helicobacter pylori* (*H. pylori*) infection in Asia [2]. The association between intestinal metaplasia, non-cardiac gastric cancer, and *H. pylori* infection has been confirmed [3]. The pathogenesis is defined by the gastric precancerous cascade [4]: normal gastric mucosa →→ superficial gastritis (later renamed non-atrophic gastritis, NAG) →→ multifocal atrophic gastritis (MAG) without intestinal metaplasia →→ intestinal metaplasia of the complete (small intestinal) type →→ intestinal metaplasia of the incomplete (colonic) type →→ low-grade dysplasia (low-grade noninvasive neoplasia) →→ high-grade dysplasia (high-grade noninvasive neoplasia) →→ invasive adenocarcinoma. Studies have shown that the eradication of *H. pylori* can reduce the incidence of gastric cancer from 63 per 100,000 person-years to 14.8 per 100,000 person-years in the commonly infected population, which is close to the incidence of 1127 per 100,000 person-years in the non-infected population. However, in the severe atrophic gastritis population, the incidence of gastric cancer in the uninfected and *H. pylori*-eradicated patients is still 75.9 per 100,000 person-years and 1.163 per 100,000 person-years, respectively [5]. In patients with intestinal metaplasia or dysplasia, eradication of *H. pylori* does not reduce the risk of gastric cancer [6, 7]. Therefore, Correa et al. believe that multifocal atrophic gastritis is the first true step in the gastric precancerous cascade [4]. However, there is a lack of specifically recognized drugs for the treatment of atrophic gastritis and intestinal metaplasia. COX inhibitors, nonsteroidal anti-inflammatory drugs (NSAIDs), Rebamipite, moluodan, and antioxidant vitamins have been suggested to be potentially effective against atrophic gastritis and intestinal metaplasia, but the quality of evidence is low. Currently, the eradication of *H. pylori* and endoscopic monitoring are the only accepted cost-effective coping strategies [6, 7].

With the widespread use of gastroscopy and pepsinogen treatment, increasing numbers of studies have shown that Chinese herbal medicine and Chinese patent medicine may be effective for atrophic gastritis and intestinal metaplasia; unfortunately, the overall research quality is low. Elian Granules is a Chinese patent medicine used in our hospital which has been in clinical use for over 20 years. It is composed of zedoary turmeric, coptis, codonopsis, salvia, angelica, dandelion, *Oldenlandia diffusa*, rhizoma atractylodis, raw licorice, pinellia, tangerine peel, and tuckahoe and has the effects of clearing heat, promoting blood circulation, and invigorating the spleen. Our preliminary studies have shown that Elian Granules can not only improve the clinical symptoms of patients with atrophic gastritis with or without intestinal metaplasia, but also may reduce gastric mucosal atrophy or intestinal metaplasia in some patients [8, 9]. The results of animal experiments have shown that Elian Granules can reverse the heterogeneity of gastric mucosal epithelial cells and reduce polyploidy and the DNA content in model rats. This reversal may be achieved by promoting the protein expression of the Fas gene and inhibiting that of Bcl-2, leading to the induction of apoptosis in gastric mucosal epithelial cells and an increased ratio of superoxide dismutase to malondialdehyde [10–12].

### Objectives {7}

The purpose of this study is to evaluate the efficacy and safety of Elian Granules in the treatment of chronic atrophic gastritis. The efficacy endpoint of the study is carcinogenesis. The safety endpoint will be determined by the number of (treatment-related) adverse events.

### Trial design {8}

**Fig. 1:**
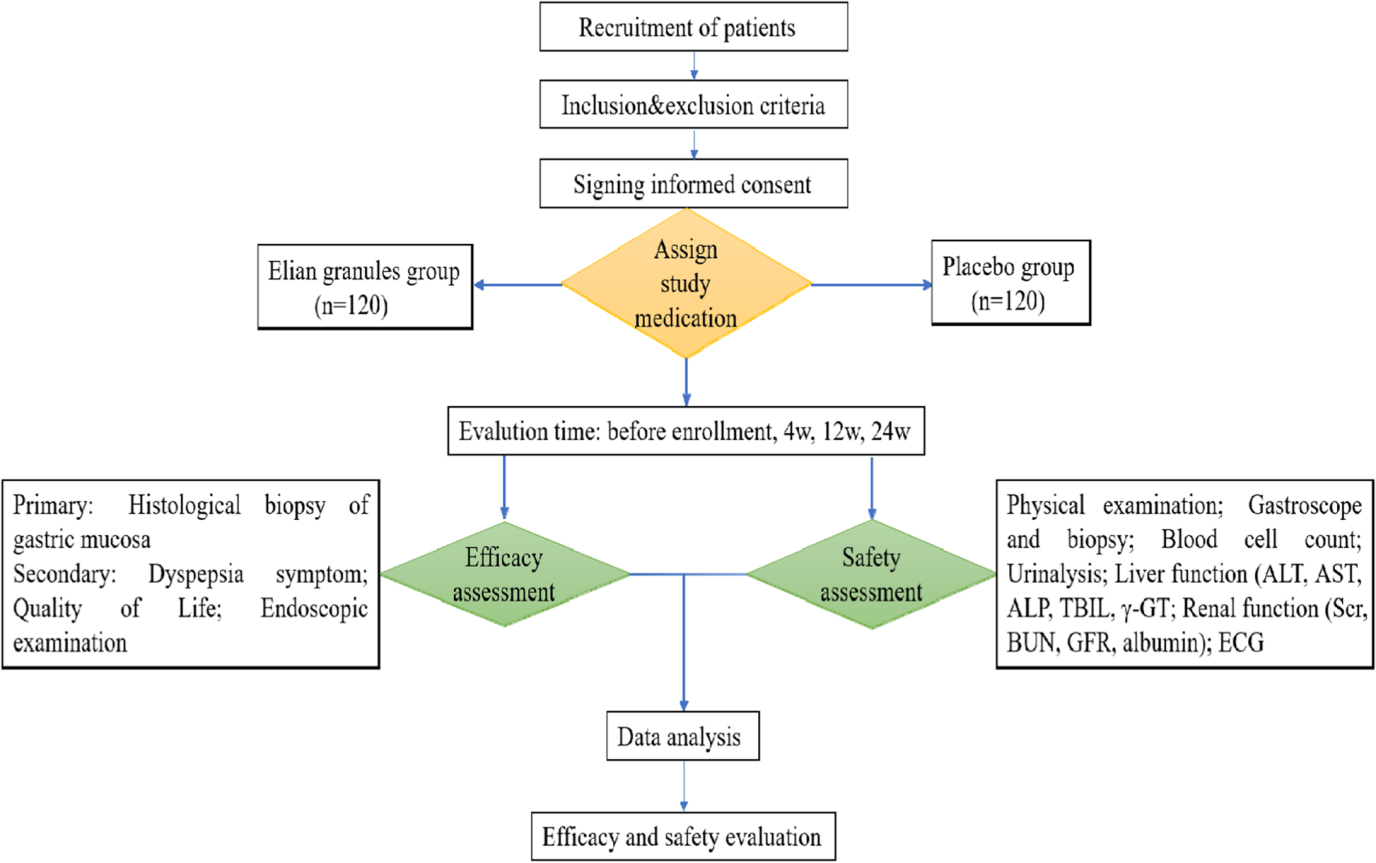
Study flow chart

The study is a randomized, double-blind, placebo-controlled, multicenter clinical superiority trial. The patient allocation ratio is 1:1. Figure 1 shows the flow chart of this trial.

## Methods: participants, interventions, and outcomes

### Study setting {9}

**Fig. 2:**
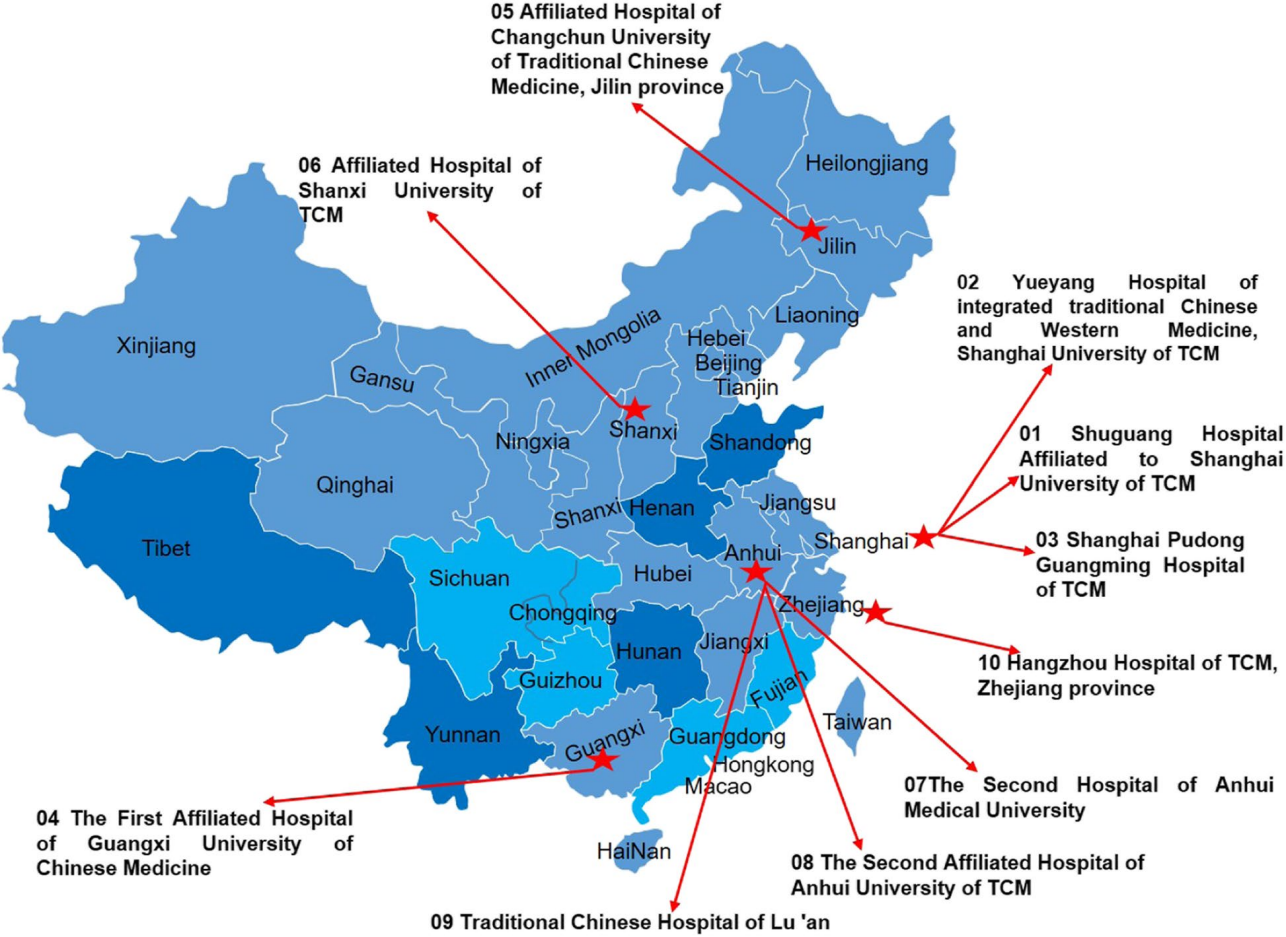
Hospital location

Two hundred forty patients will be enrolled from 10 hospitals in 6 provinces in China, including (1) Shuguang Hospital affiliated with Shanghai University of Traditional Chinese Medicine; (2) Yueyang Hospital of integrated traditional Chinese and Western Medicine, Shanghai University of Traditional Chinese Medicine; (3) Shanghai Pudong Guangming Hospital of Traditional Chinese Medicine; (4) The First Affiliated Hospital of Guangxi University of Chinese Medicine; (5) Affiliated Hospital of Changchun University of Traditional Chinese Medicine; (6) Affiliated Hospital of Shanxi University of Traditional Chinese Medicine; (7) The Second Hospital of Anhui Medical University; (8) The Second Affiliated Hospital of Anhui University of Traditional Chinese Medicine; (9) Traditional Chinese Hospital of Lu’an; and (10) Hangzhou Hospital of Traditional Chinese Medicine, and are considered for inclusion if they meet the criteria as defined below, as shown in Fig. 2.

### Eligibility criteria {10}

#### Diagnosis

Diagnosis will be made in accordance with the Chinese Consensus on Chronic Gastritis (2017, Shanghai) formulated by the Society of Digestive Diseases, Chinese Medical Association [13]. The diagnostic criteria for chronic atrophic gastritis are as follows.

#### Clinical manifestations and physical signs

Chronic gastritis has no specific clinical manifestations, such as epigastric pain, distention, belching, acid regurgitation, nausea, vomiting, and loss of appetite. The presence or absence of dyspepsia and its severity do not correlate significantly with the classification of chronic gastritis, its endoscopic features, or gastric mucosal histopathologic staging. Most patients have no obvious physical signs, although sometimes mild upper abdominal tenderness or discomfort may occur.

#### Endoscopic and histopathological diagnosis

**Fig. 3:**
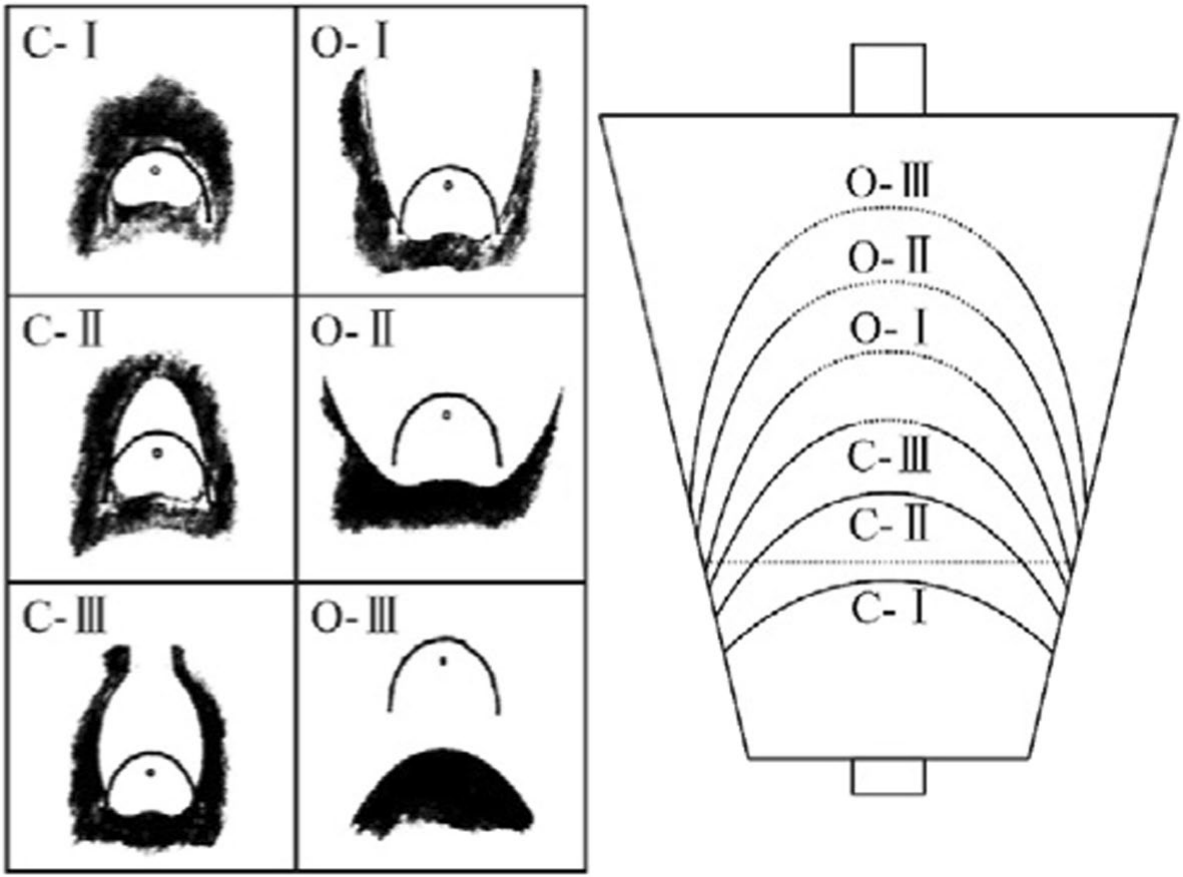
Extension of the atrophic border and patterns of endoscopic gastric atrophy as classified by Kimura and Takemoto

The endoscopic findings of chronic atrophic gastritis include mixed red and white coloration of the mucosa dominated by white mucosa, flat or even absent folding, exposure of part of the mucosal blood vessels, and a possible association with mucosal granules or nodules. The diagnosis of chronic atrophic gastritis includes both endoscopic and pathologic diagnoses, while atrophy diagnosed under white-light endoscopy has a low concordance with a pathologic diagnosis. The final diagnosis should be based on the pathologic diagnosis. The endoscopic gastric pattern is defined according to the Kimura-Takemoto classification (Table 1), based on the location of the endoscopic atrophic border (Fig. 3). The histopathological diagnosis refers to the pathological diagnostic criteria of chronic gastritis in China and the visual analog scale [13]. The severity of atrophic gastritis is defined according to OLGA and OLGIM [14, 15].

**Table 1:** Kimura-Takemoto classification

Type	Classification	Definition
Closed Type	C-1	Atrophy visible in the antrum but not in the corpus
	C-2	Atrophy visible parabolically above the angulus and below the middle of the stomach on the lesser curvature
	C-3	Atrophy visible parabolically above the middle of the stomach on the lesser curvature
Opened Type	O-1	The atrophic border lies between the lesser curvature and the anterior wall
	O-2	The atrophic border lies on the anterior wall
	O-3	The atrophic border lies between the anterior wall and the greater curvature

#### Inclusion criteria

Patients must meet the following criteria to be eligible for enrollment:

1. Patients aged 18–70 years, male or female
2. Patients who meet the diagnostic criteria of chronic atrophic gastritis and who test negative for *Helicobacter pylori* (13C or 14C urea breath test is required)
3. Patients with a pathological stage of OLGA-II/III. Two experts from the Department of Pathology will perform the diagnosis (pathological film reading), with consensus on the pathological conclusion
4. Patients who have voluntarily agreed to participate and who have signed informed consent

#### Exclusion criteria

If the patients meet any of the following criteria at the screening visit, they will not be eligible for the study:

1. Patients with peptic ulcer, severe dysplasia of the gastric mucosa, or suspected pathological diagnosis of malignant transformation
2. Patients with severe diseases of the digestive system or a history of abdominal surgery
3. Pregnant or lactating women
4. Patients with severe heart and lung diseases, malignant tumors, severe diabetes mellitus, and chronic liver and kidney dysfunction and those in whom the ALT and AST levels indicative of deteriorated liver function are more than 1.5 times the upper limit of normal and in whom there is renal function damage (BUN > 1.2 times of the upper limit of normal; Cr > the upper limit of normal)
5. Patients in whom the PLT is < 1.5 times that of the lower limit of normal
6. Disabled patients (blind, deaf, dumb, mental disorder, physical disability) as prescribed by law
7. Alcoholics
8. Those who are known to be allergic to two or more types of drugs or the ingredients of the drug and have allergic constitutions
9. Patients who have participated in other clinical trials during the previous 4 weeks. Patients who have taken Weifuchun, Moluodan, and folic acid within the previous 2 weeks
10. Patients who, in the opinion of the investigator, are likely to be lost to follow-up or are not suitable for inclusion
11. Patients who withdraw their informed consent

### Who will take informed consent? {26a}

Patients with chronic atrophic gastritis confirmed by histopathology will be screened for eligibility to participate in this study based on the abovementioned criteria. After the patient has been assessed as eligible, he/she will receive the initial study information. After at least 2 weeks of reflection, patients are invited to meet with the research physician to discuss any remaining questions and sign the informed consent.

### Additional consent provisions for the collection and use of participant data and biological specimens {26b}

No additional consent provisions.

## Interventions

### Explanation for the choice of comparators {6b}

1. Because there is no specific drug for the treatment of chronic atrophic gastritis with or without intestinal metaplasia, it is ethical to conduct this study as placebo controlled
2. A 6-month course of treatment is appropriate and will not delay the patient’s condition
3. A statistically significant minimum number of cases will be used in this study
4. Patients will be informed about the content of the placebo treatment and will sign informed consent
5. The safety of the placebo will be ensured by endoscopic review after treatment
6. In order to protect patients, the protocol provides that the investigator may withdraw the patients if the patient’s condition changes during the trial and other effective treatments are needed
7. It is helpful to objectively evaluate the safety and effectiveness of Elian Granules with controlled evaluations

### Intervention description {11a}

Elian Granules and placebo are produced by the Jiangyin Tianjiang Pharmaceutical Co. Ltd., China, for clinical research only (Approval No. 2006338). The placebo consists of 36% maltodextrin, 11% lemon yellow intermediate, 15% sunset yellow intermediate, 18% caramel intermediate, 4% bittering agent, 1% traditional Chinese medicine essence, and 10% lactose.

Although the placebo is similar to Elian Granules in color and smell, it is not the same. To make a blind test possible, all the drugs will be concealed in uniform, sealed, and opaque packages with the same labels that contain the drug name, the approval number of the pill, functions, usage, dosage, storage conditions, expiration dates for use, and the manufacturer’s name. The drugs will be administered by an independent clinical assistant in each center, who will take responsibility for the drug’s distribution, storage, and return. All drugs will be registered in the “drug-use record for clinical trials,” including information on the date of release of the trial drugs, the names of the subjects, and the quantities of drugs provided and retrieved. According to the intervention plan, there will be 6 medium packages contained in each large package and 112 small bags of drugs (12.1 g/bag) (Table 2) in each medium package. The patients will be randomly divided into the Elian Granules treatment group and the placebo control group. All drugs will be taken with hot water half an hour after meals, two small bags each time, twice a day for 24 weeks. Follow-up visits will be arranged at 4, 12, and 24 weeks after treatment. After the clinical study, the patients will receive routine treatment.

**Table 2:** Components and dose of Elian Granules

Formula name	Chinese name	English name	Weight (g)	Weight (%)
Elian Granules	Ezhu	Zedoary turmeric	0.275	2.27
	Huanglian	Coptis	0.125	1.03
	Dangshen	Codonopsis	2.25	18.59
	Danshen	Salvia	0.8	6.61
	Danggui	Angelica	1.625	13.43
	Pugongying	Dandelion	1.475	12.19
	Baihuasheshecao	Oldenlandia diffusa	1.075	8.88
	Baizhu	Rhizoma atractylodis	1.65	13.64
	Shenggancao	Raw licorice	0.275	2.27
	Banxia	Pinellia	1	8.26
	Chenpi	Tangerine peel	0.55	4.55
	Fuling	Tuckahoe	1	8.26
	Total		12.1	100

### Criteria for discontinuing or modifying allocated interventions {11b}

Patients will be able to leave the study at any time for any reason if they wish to do so without any consequences. The patient’s participation in this study can also be ended by the investigator if the patient is uncooperative and/or does not attend study visits. The patient data that have been collected up to that point will be included in the analysis. This study will be prematurely ended in the event of multiple adverse events. The criteria for study termination include any suspected unexpected serious adverse reaction (SUSAR) or serious adverse event (SAE) based on an allergic reaction and clear allergic or iatrogenic effects in two or more patients.

### Strategies to improve adherence to interventions {11c}

During the clinical trial, to ensure the compliance of the subjects, it is important that they fully understand the significance of the trial, the importance of taking the medicine on time, and the necessity for follow-up. The subjects will be required to take the medicine according to the regulations and to fill in the patient diary card on time. The drug counting method (medication compliance = (actual dosage/required dosage) × 100%) combined with inquiry will be used to judge the compliance of the subjects taking the test drug. These issues will be fully and patiently explained to the participants, who will be asked to take the medicine and attend the follow-up as required.

### Relevant concomitant care permitted or prohibited during the trial {11d}

In addition to prescribed medication, the following traditional Chinese and Western medicines, as well as other factors that could potentially affect chronic atrophic gastritis, will be prohibited during the trial period. If a participant is found to be taking these drugs, they will be withdrawn from the study.

1. The use of other drugs, such as folic acid, selenium, yeast, vitamin C, and vitamin E, will not be allowed [7]
2. The use of Chinese patent medicines that may have curative effects, including the reversal or slowing of atrophy progression, will also not be allowed. These medicines include Weifuchun, Jinghuaweikang capsules, moluodan, Dalitong granules, Qizhiweitong granules, bilingweitong granules, Wenweishu capsules, Xiaojianzhong capsules, Yangweishu capsules, Zhizhukuanzhong capsules, and Weisu granules, among others, as well as any Chinese herbal decoction
3. No any acupuncture therapy for treating chronic atrophic gastritis will be allowed during the trial

It will also not be allowed to use any drugs which have a therapeutic effect on gastritis, such as PPIs and/or prokinetic agents during the study.

Drugs or other treatments that are required to be taken continuously for the treatment of complex diseases can be used under the guidance of researchers. Any drugs used during the trial must be recorded and explained in detail in the study cases, including the name of the disease, drugs, dosage, and usage, so that they can be analyzed and reported when summarized.

### Provisions for post-trial care {30}

The sponsor shall bear the cost of treatment and corresponding economic compensation for the subjects who suffer any damage or death related to the trial. The sponsor shall provide legal and economic guarantees to the researcher, except in the event of issues caused by medical malpractice.

### Outcomes {12}

#### Primary outcome

The histological assessment of gastric mucosal biopsies is the primary outcome of the study. OLGA and OLGIM staging will be used for pathological evaluation. The evaluation will be performed both before and after 24 weeks of administration of the treatment and any histopathological changes in the gastric mucosa will be compared between the two groups before and after 24 weeks of treatment. The outcomes will be divided into progressive, stable, and improved according to the OLGA/OLGIM stage after treatment. An increase in staging grade will represent progression, an unchanged staging grade will represent stability, and a reduction in staging grade will be considered an improvement.

#### Secondary outcomes

##### Endoscopic examination

Gastric atrophy before and after treatment will be assessed in terms of the Kimura-Takemoto classification.

##### Dyspepsia symptoms

A symptom score system will be used to evaluate the improvement in dyspepsia (Table 3). In the symptom score system, the score will be determined according to the severity, duration, and frequency of symptoms. The symptom scores will be recorded before administration and after 4 weeks, 12 weeks, and 24 weeks. The overall score represents the sum of all symptom scores. The dyspepsia symptom scores and the changes in each index value before and after treatment will be compared between groups. The treatment effect index (TEI) will be calculated as (pre-treatment integral − post-treatment integral)/pre-treatment integral × 100%. The curative effect classification is as follows: TEI ≥ 90% indicates clinically cured; 90% > TEI ≥ 70% indicates marked efficacy; 70% > TEI ≥ 30% indicates efficacy; TEI < 30% indicates invalid.

**Table 3:** The symptom score system for chronic atrophic gastritis

Symptom	Score	Degree	Grading system
Epigastric pain	1	None	-
	2	Mild	< 2 times a week
	3	Moderate	≥ 3 times a week, not every day
	4	Severe	Every day, intermittently
	5	Extreme severe	Every day, almost continuously
Epigastric distention	1	None	-
	2	Mild	< 2 times a week
	3	Moderate	≥ 3 times a week, not every day
	4	Severe	Every day, intermittently
	5	Extreme severe	Every day, almost continuously
Epigastric discomfort	1	None	-
	2	Mild	< 2 times a week
	3	Moderate	≥ 3 times a week, not every day
	4	Severe	Every day, intermittently
	5	Extreme severe	Every day, almost continuously
Early satiety	1	None	-
	2	Mild	< 2 times a week
	3	Moderate	≥ 3 times a week, not every day
	4	Severe	Every day, intermittently
	5	Extreme severe	Every day, almost continuously
Heartburn	1	None	-
	2	Mild	< 2 times a week
	3	Moderate	≥ 3 times a week, not every day
	4	Severe	Every day, intermittently
	5	Extreme severe	Every day, almost continuously
Acid regurgitation	1	None	-
	2	Mild	< 2 times a week
	3	Moderate	≥ 3 times a week, not every day
	4	Severe	Every day, intermittently
	5	Extreme severe	Every day, almost continuously
Belching	1	None	-
	2	Mild	< 2 times a week
	3	Moderate	≥ 3 times a week, not every day
	4	Severe	Every day, intermittently
	5	Extreme severe	Every day, almost continuously
Abdominal discomfort	1	None	-
	2	Mild	< 2 times a week
	3	Moderate	≥ 3 times a week, not every day
	4	Severe	Every day, intermittently
	5	Extreme severe	Every day, almost continuously
Total symptom score	Equal to the sum of the above scores		

##### Quality of life

Quality of life will be assessed by a 12-item Short-Form Health Survey (SF-12) (Table 4). The scale items include eight dimensions: general health (GH), physical functioning (PF), role physical (RP), body pain (BP), role emotional (RE), mental health (MH), vitality (Vt), and social function (SF). Scores will be calculated as percentages. The first four dimensions belong to the physical component summary (PCS) level, while the last four dimensions belong to the mental component summary (MCS) level. We will compare the quality of life and the value of each index between the two groups.

**Table 4:** Twelve-item Short-Form Health Survey (SF-12)

Items	Score	Grading system
In general, would you say your health is?	1	Excellent
	2	Very good
	3	Good
	4	Fair
	5	Poor
The following two questions are about activities you might do during a typical day. Does your health now limit you in these activities? If so, how much?		
Moderate activities, such as moving a table, pushing a vacuum cleaner, bowling, or playing golf?	1	Yes, limited a lot
	2	Yes, limited a little
	3	No, not limited at all
Climbing several flights of stairs?	1	Yes, limited a lot
	2	Yes, limited a little
	3	No, not limited at all
During the past 4 weeks have you had any of the following problems with your work or other regular activities as a result of your physical health?		
Accomplished less than you would like?	1	Yes
	2	No
Are limited in the kind of work or other activities?	1	Yes
	2	No
During the past 4 weeks, have you been limited in the kind of work you do or other regular activities as a result of any emotional problems (such as feeling depressed or anxious)?		
Accomplished less than you would like?	1	Yes
	2	No
Cannot do work or other activities as carefully as usual?	1	Yes
	2	No
During the past 4 weeks, how much did pain interfere with your normal work (including both work outside the home and housework)?	1	Not at all
	2	A little bit
	3	Moderately
	4	Quite a bit
	5	Extremely
The next three questions are about how you feel and how things have been during the past 4 weeks. For each question, please give the one answer that comes closest to the way you have been feeling. How much of the time during the past 4 weeks?		
Have you felt calm and peaceful?	1	All of the time
	2	Most of the time
	3	A good bit of the time
	4	Some of the time
	5	A little of the time
	6	None of the time
Do you have a lot of energy?	1	All of the time
	2	Most of the time
	3	A good bit of the time
	4	Some of the time
	5	A little of the time
	6	None of the time
Have you felt downhearted and blue?	1	All of the time
	2	Most of the time
	3	A good bit of the time
	4	Some of the time
	5	A little of the time
	6	None of the time
During the past 4 weeks, how much of the time has your physical health or emotional problems interfered with your social activities (like visiting with friends, relatives, etc.)?	1	All of the time
	2	Most of the time
	3	A good bit of the time
	4	Some of the time
	5	A little of the time
	6	None of the time

### Safety outcomes

The following tests will be performed on all subjects during the study: physical examination (temperature, respiration, heart rate, blood pressure, height, and weight); gastroscope and biopsy; complete blood cell count; urinalysis; liver function (ALT, AST, alkaline phosphatase [ALP], serum total bilirubin [TBIL], and γ-glutamyl transpeptidase (γ-GT)); renal function (Cr, BUN) and glomerular filtration rate (GFR); and electrocardiography (ECG). In addition, a urine pregnancy test will be carried out for female patients of childbearing age.

### Participant timeline {13}

Table 5 shows the participant timeline.

**Table 5:** Participant timeline

Visit in doctor’s office	Enrollment	Baseline	Post-allocation		Close-out
	Visit 1	Visit 2	Visit 3	Visit 4	Visit 5
Time point	-4W-0day	0day	4W±3day	12W±3day	24W±3day
Information collection					
Demographic information	×				
Physical examination	×		×	×	×
Informed consent	×				
Medical history	×				
Past history	×				
Concomitant treatment	×		×	×	×
Review selection	×				
Allocation		×			
Interventions					
Treatment group			×	×	×
Control group			×	×	×
Assessments					
Clinical sign and symptom score	×		×	×	×
Chinese medicine diagnosis	×				
Endoscope					
Kimura-Takemoto classification	×				×
Biopsy	×				×
13C or 14C breath test	×				
SF-12	×				×
Complete blood cell count	×				×
Liver function	×				×
Renal function	×				×
Urinalysis	×				×
ECG	×				×
Urine pregnancy test	×				
Adverse events			×	×	×
Efficacy determination					×
Others					
Drug distribution		×	×	×	
Drug recycling and count			×	×	×
Patient record card release		×			
Patient record card recycling					×
Check patient record card and conduct clinical evaluation			×	×	×
Summary					×

### Sample size {14}

The design is a randomized, double-blind, double-dummy, parallel-controlled, multicenter clinical trial. The sample ratio of the treatment group and the control group is 1:1. Based on the preliminary results of clinical research [8, 9], the expected efficacy of Elian Granules on chronic atrophic gastritis is 85%, with 63.3% of the therapeutic effect of Weifuchun used as the placebo effect. According to relevant references [16, 17] and the above data, two independent proportions superiority power analysis is used to estimate the sample size based on *α* = 0.05, *β* = 0.10, and power = 0.90 by Pass 11.0. It was estimated that no fewer than 95 subjects should be enrolled in each group. Allowing for a 20% drop-out, we, therefore, propose the recruitment of a total sample size of 220 patients. Referring to the phase II clinical trial, the planned total sample size is 240 cases in two groups. In terms of competition, each center should provide no fewer than 20 cases.

### Recruitment {15}

Recruitment of patients will take place in 9 tertiary hospitals and 1 secondary hospital in 6 provinces and cities in China, which can ensure the enrollment of sufficient subjects.

## Assignment of interventions: allocation

### Sequence generation {16a}

Central randomization and competitive enrollment will be used. SAS software will be used for randomization by providing the number of seeds and setting the block size to 4, thus generating the random arrangement table. Subjects will be competitively enrolled in 10 hospitals.

### Concealment mechanism {16b}

The random arrangement table generated by block randomization will be sealed as confidential data. According to the random arrangement table, the drugs are blinded by personnel unrelated to the trial, and the clinical research centers will administer the drugs according to the assigned drug number and the order of case selection. The blind code will be in triplicate and sealed in the office of the National Drug Clinical Trial Institution of Shuguang Hospital affiliated with Shanghai University of Traditional Chinese Medicine, Office of Clinical Trial Institution of Cooperation Unit and Statistical Unit. Each coded trial drug will have corresponding emergency letters, allowing to the blind be broken in the case of emergency. The emergency letters will be stored in each trial unit and collected at the end of the trial.

### Implementation {16c}

The allocation sequence will be generated by a statistician independent of the data management statistical analysis performed in this trial. The recruitment and intervention of subjects will be completed by the researchers of this project in each clinical research center.

## Assignment of interventions: blinding

### Who will be blinded {17a}

Subjects, researchers, and data analysts will be blinded.

### Procedure for unblinding if needed {17b}

Each drug number will be provided with an emergency letter, in which the corresponding drug group is sealed. In case of a medical emergency (for example, if the subject requires rescue), it can be opened. Should it be opened, the letter should be signed and dated by the reader, and the reason for opening will be recorded. In the event of an emergency, researchers and project leaders should attend and record the reason, time, and place of unblinding in detail and sign. The clinical trial team leader and the clinical supervisor will be informed timeously after unblinding. The case data will be kept intact. The emergency letter will be sent to each clinical research unit with the corresponding drug number and will be taken back after the trial.

## Data collection and management

### Plans for assessment and collection of outcomes {18a}

The project will use electronic data capture (EDC) to collect and manage data and the REDCap system for synchronous data input. Before data entry, the data administrator will conduct EDC and REDCap system training to facilitate understanding of the overall situation, as well as mastering the data entry and export methods. The data manager and the principal researchers will work together to write the relevant documents, and the data manager will build and test the electronic case report form (eCRF). The supervisor will oversee the adherence of the trial to the trial plan and data entry, checking whether the data entry is synchronous, correct, complete, and consistent with the original data. Should errors and omissions occur, the researcher will be asked to correct them timeously. Only researchers and supervisors will have access to the subjects’ medical records, and they will sign a confidentiality agreement. Data managers, researchers, supervisors, and statisticians will conduct blind reviews and prepare a report on the quality of the data management. After the blind review and confirmation of the accuracy of the database, the database will be locked. After the data are locked, they will be handed over to the statisticians, and SAS 6.12 statistical analysis software will be used for statistical analysis in a semi-blind way, and the statistical analysis report will be written. After analysis, the medical records of the subjects will be kept in the data files of the drug clinical trial institution.

### Plans to promote participant retention and complete follow-up {18b}

The patients will receive extensive information about the study setup and requirements during the recruitment process. The importance of completing the follow-up will be stressed. Patients will be allowed to stop at any time during the study and will not be obliged to give a reason for discontinuation. All patients will be reminded throughout the study to fill out the questionnaires during the study visits. Throughout the follow-up period, the researchers will check responses and, if necessary, will contact the patients for completion of their follow-up.

### Data management {19}

**Fig. 4:**
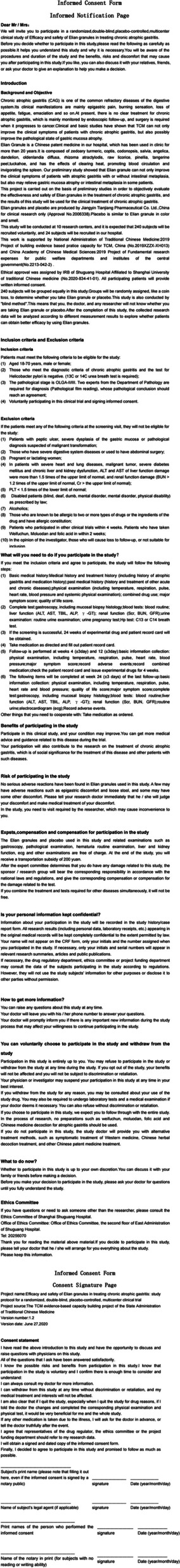
Model consent form

An eCRF meeting GCP criteria will be used to collect patient data. The data entered through the REDCap system will be stored in the research folder on the protected research server and will be backed up regularly (once every 3 months). The research data (including informed consent) will be filled in, reviewed, and signed by the supervisor and the person in charge of each unit according to the data management requirements of this protocol. After the agency office is sealed, the original copy together with the original medical records will be submitted to the experimental unit for retention, one to the data management and statistical analysis unit, and one to the Shuguang Hospital affiliated with Shanghai University of Traditional Chinese Medicine. The researcher will keep all the research data, including the confirmation of all the participants (and can effectively check different records, such as the original records of the hospital), all the original informed consent forms signed by the patients, all the research medical records, and detailed records of drug distribution (Fig. 4). Shuguang Hospital affiliated with Shanghai University of Traditional Chinese Medicine will retain the clinical trial data for 5 years after the termination of the clinical trial.

### Confidentiality {27}

Research data will be stored using each participant’s research identification code. The key to the identification code list will be available only to the research team during the study and will be recorded and protected according to the research guidelines after the study is completed. Details of the patients’ identities will not be reported in the publication.

### Plans for collection, laboratory evaluation, and storage of biological specimens for genetic or molecular analysis in this trial/future use {33}

The biopsy samples will be kept in the Pathology Department of the hospital. Blood samples will be kept in the hospital laboratory. All samples will be preserved and destroyed according to the relevant hospital regulations.

## Statistical methods

### Statistical methods for primary and secondary outcomes {20a}

Full analysis set (FAS): this refers to the ideal subject set that is as close as possible to the principle of intention to analyze (including all subjects who are randomized into the group and receive at least one treatment). For the estimation of missing values of major variables, if the case data of all treatment processes cannot be observed, they will be carried forward to the missing data of the test, ensuring that the number of subjects evaluated for efficacy at the end is consistent with that at the beginning of the trial.

Per protocol set (PPS): the main variables are able to be determined for all patients who meet the experimental treatment protocol, have good compliance, use 80–120% of the experimental drugs, and complete the CRF requirements, with no missing baseline variables and no major violation of the experimental protocol.

Safety analysis set (SS): all subjects who received at least one treatment after randomization.

The main variables and comprehensive efficacy analysis constitute the full analysis set and the per-protocol set; demographic and other baseline characteristics will be analyzed using the full analysis set; the safety set will be selected for safety evaluation.

Baseline equilibrium analysis will be conducted in FAS.

The equilibrium analysis of the basic value is aimed at the basic demographic characteristics, vital signs, and curative effect-related indicators, among others, on enrollment into the group, to explain whether the basic characteristics of the two groups are comparable. Among them, the quantitative indicators (such as age, course of disease, temperature, pulse, respiration, systolic blood pressure, diastolic blood pressure, and TCM syndrome score) of the two groups will be listed, including the number of cases, means, standard deviations, medians, maxima, and minima. The two groups will be compared by ANOVA and the Kruskal-Wallis rank sum test. The frequencies and percentages of qualitative indicators (such as nationality, marital status, heart rate, history, allergy history, single symptom) will be listed and compared by *χ*^2^ test/precise probability method/rank sum test.

Analysis of efficacy will be carried out in both FAS and PPS. According to the OLGA/OLGIM staging changes after treatment, the efficacy of CAG will be divided into progressive, stable, and improved. The numbers and percentages of cases will be calculated according to the efficacy rate. The CMH chi-square test stratified by center will be used to compare the two groups, and the 95% confidence interval of the rate difference between the two groups will be calculated. The curative effect on clinical symptoms will be calculated by the number and percentage of cases classified as cured, markedly effective, effective, and ineffective. The CMH chi-square test stratified by center will be used to compare the two groups. For the assessment of the changes in mucosal histology before and after treatment, changes after treatment will be evaluated relative to the baseline, and the number of cases, means, standard deviations, medians, maxima, and minima will be calculated and compared within the groups using paired *t*-test/signed-rank tests and compared between the two groups by analysis of variance. A covariance analysis model will be used to compare the difference between the two groups after treatment relative to the baseline. In the model, the baseline will be taken as the covariate, and the effects of grouping and center will be considered. The least-squares mean and 95% confidence interval of the difference between the groups are calculated based on this model.

The safety analysis will be carried out in SS. Adverse events will be recorded according to their occurrence and the numbers and percentages of cases will be listed and compared using the chi-square test/Fisher exact probability method. During the treatment, all adverse events will be classified and coded according to the common term evaluation standard for adverse events (CTCAE 4.0). The number of cases, times, and incidence rates will be calculated according to the system organ classification and standard name after coding, and the details will be described in a list. Laboratory examinations related to safety: all completed laboratory examination items will be listed in the form of a cross table before and after treatment (according to their clinical significance), and abnormal results obtained after treatment will be listed. Vital signs: changes in physical signs (body temperature, heart rhythm, respiration, systolic blood pressure, diastolic blood pressure) before and after treatment will be described and the number of cases, means, standard deviations, medians, maxima, and minima will be calculated and compared using ANOVA, with the abnormal results listed after treatment.

### Interim analyses {21b}

There are no interim analyses planned.

### Methods for additional analyses (e.g., subgroup analyses) {20b}

There are no subgroup analyses planned.

### Analytical methods for handling protocol non-adherence and any statistical methods for handling missing data {20c}

The drug counting method combined with inquiry will be used to judge the compliance of the subjects taking the test drug. Medication compliance = (actual dosage/required dosage) × 100%. Good compliance would be represented by 80~120%. The study will be evaluated by intention analysis, which can minimize the missing data.

### Plans to give access to the full protocol, participant-level data, and statistical codes {31c}

The data sets used in the current study can be provided by the corresponding authors on reasonable request.

## Oversight and monitoring

### Composition of the coordinating center and trial steering committee {5d}

This is a multicenter study in 10 hospitals. The daily operation of the study will consist of the following personnel:

- Principal investigator: responsible for the supervision of the experiment and the medical responsibility of the subjects
- Data administrator: responsible for data acquisition, data quality, and safety
- Research coordinator: responsible for trial registration and coordinating the research of each center
- Supervisor: responsible for monitoring the safety status and research progress of subjects
- Assistant investigator: responsible for the identification of potential subjects, obtaining informed consent, and ensuring follow-up according to the protocol

The research group will meet once a month. There will be no trial steering committee or stakeholder and public participation group.

### Composition of the data monitoring committee and its role and reporting structure {21a}

Shuguang Hospital affiliated with Shanghai University of Traditional Chinese Medicine will appoint a third party as a supervisor to supervise the safety status and research progress of the subjects. The supervisor will contact the research center regularly, including visiting the research center. The degree, nature, and frequency of the visit will depend on the consideration of the research project and/or endpoint, the research purpose, the complexity of the research design, and the speed of the selection of the subjects.

During the contact, the inspector will:

1. Check and evaluate the progress of the study
2. Review the research data collected
3. Check the original data
4. Identify problems and propose solutions

This is to ensure that:

1. The data are reliable, accurate, and complete
2. The safety and rights of the subjects are protected

The study is conducted in accordance with the currently approved protocol, GCP, and all current registration regulations.

### Adverse event reporting and harms {22}

The safety of subjects during the study period is very important, and every adverse event will be recorded in detail on the adverse event form (AEF). Adverse events (AE) include any new disease, aggravation of the original disease, and complications related or unrelated to treatment. Any fatal, life-threatening, disabling, or serious event that results in hospitalization or long-term hospitalization will be considered to be an SAE. All these data will be recorded on the AEF along with the corresponding treatment methods and will be reported to the China Food and Drug Administration (CFDA), the Provincial Food and Drug Administration, and the ethics committee within 24 h. Adverse events will be reported to the other research centers at the same time. The severity of acute events will be divided into three grades: mild, moderate, and severe. A mild event represents a small amount of physical discomfort, with no intervention necessary, and having no effect on the study. Subjects experiencing severe events will, for their own safety, be withdrawn from the study. In the case of SAE, the researchers will be able to confirm the treatment allocation of the subjects through emergency unblinding. Any AE related to the trial drug will be treated free of charge. In addition, all adverse events will be recorded and tracked until they have been properly resolved or have stabilized. Any abnormal physical or chemical indices recorded after the medication will be reexamined to ensure that they return to normal or become stable.

### Frequency and plans for auditing trial conduct {23}

In order to meet the requirements of the GCP and current regulations, Shuguang Hospital affiliated with Shanghai University of Traditional Chinese Medicine will be able to audit the quality of the study at its own discretion. This audit/review can be carried out at any time during or after the study. If such an audit/review is conducted, the researcher and the research center should agree to allow the auditor/reviewer direct access to all relevant documents and arrange time to discuss the findings and related issues with the supervisor/reviewer.

### Plans for communicating important protocol amendments to relevant parties (e.g., trial participants, ethical committees) {25}

The competent authority will be notified of all substantial amendments. Non-substantial amendments will be recorded and filed. In the event of amendments causing concern or affecting participants in any way, they will be informed of the changes. If needed, additional consent will be requested and registered. Also, online trial registries will be updated accordingly.

## Dissemination plans {31a}

The results of this study will be fully disclosed in the journal. Both positive and negative results will be reported.

## Discussion

This study is designed to evaluate the efficacy and safety of Elian Granules in a randomized, double-blind, placebo-controlled, multicenter manner. This trial may not only provide evidence for a phase III clinical trial, but also an alternative option for the treatment of chronic atrophic gastritis (CAG).

## Trial status

Recruiting started in November 2010. The current protocol is version 1.3 of 13-9-2019. Currently (16th of July 2020), we have included 81 patients. Patient recruitment is estimated to be completed around November 2022.

## Data Availability

The datasets used and/or analyzed during the current study will be made available from the corresponding author upon reasonable request.

## Abbreviations

CAG: Chronic atrophic gastritis
NAG: Non-atrophic gastritis
MAG: Multifocal atrophic gastritis
OLGA: Operative link for gastritis assessment
OLGIM: Operative link for gastric intestinal metaplasia assessment
ALT: Alanine aminotransferase
AST: Aspartate aminotransferase
BUN: Blood urea nitrogen
Cr: Creatinine
PLT: Platelet
SUSAR: Suspected unexpected serious adverse reaction
SAE: Serious adverse event
TEI: Treatment effect index
ALP: Alkaline phosphatase
TBIL: Serum total bilirubin
γ-GT: γ-Glutamyl transpeptidase
GFR: Glomerular filtration rate
ECG: Electrocardiogram
EDC: Electronic data capture
eCRF: Electronic case report form
FAS: Full analysis set
PPS: Per-protocol set
SS: Safety analysis set
TCM: Traditional Chinese Medicine
ANOVA: Analysis of variance
AE: Adverse event
AEF: Adverse event form
CFDA: The China Food and Drug Administration
